# Romantic Relationships and Mental Health During the COVID-19 Pandemic in Austria: A Population-Based Cross-Sectional Survey

**DOI:** 10.3389/fpsyg.2022.857329

**Published:** 2022-04-27

**Authors:** Benedikt Till, Thomas Niederkrotenthaler

**Affiliations:** ^1^Unit Suicide Research and Mental Health Promotion, Department of Social and Preventive Medicine, Center for Public Health, Medical University of Vienna, Vienna, Austria; ^2^Wiener Werkstaette for Suicide Research, Vienna, Austria

**Keywords:** mental health, romantic relationship, relationship satisfaction, relationship commitment, family structure, COVID-19

## Abstract

**Background:**

Previous studies suggest that romantic relationships can be beneficial to mental health, but may also be a major stressor depending on specific relationship characteristics. Studies examining the role of romantic relationship in mental health are scarce. This study aimed to investigate differences in mental health with regards to relationship characteristics.

**Methods:**

We assessed individuals’ mental health, i.e., suicidal ideation (via Beck Scale for Suicidal Ideation, BSS), depression (via Patient Health Questionnaire, PHQ-9), anxiety (Hospital Anxiety and Depression Scale, HADS), experience of psychological and physical violence, including changes in suicidal ideation and anxiety compared to before the pandemic, and relationship characteristics (i.e., relationship status, satisfaction, and commitment as well as family structure) with online questionnaires in a population-based cross-sectional study with 3,012 respondents in Austria during the COVID-19 pandemic.

**Results:**

There were small to medium–sized group differences with regards to relationship status and satisfaction (η_*p*_^2^: 0.011–0.056). Most mental health outcomes were less favorable in singles than in individuals in happy relationships, but scores for anxiety (*p* < 0.001), psychological (*p* < 0.001) and physical violence (*p* < 0.001), and the probability of experiencing an increase in anxiety compared to before the pandemic (*p* < 0.01) were lower in singles as compared to those with low relationship satisfaction. Furthermore, scores for suicidal ideation (*p* > 0.001) and psychological (*p* > 0.01) and physical violence (*p* > 0.01) were highest in individuals in relationships with low commitment and with a child living in the same household, but effect sizes were small (η_*p*_^2^: 0.004–0.015).

**Conclusion:**

During the COVID-19 pandemic, as compared to singles, mental health appeared worse in individuals with low relationship satisfaction and those in a relationship with low commitment and with a child in the household. Living in a happy relationship was associated with somewhat better mental health.

## Introduction

The relationship people have with their spouse or partner is an essential and integral factor in the lives of most individuals (Whisman and Baucom, 2012; Kazan et al., 2016). The social support individuals may receive from a spouse can be reflected in their well-being (Antonucci et al., 2001; Okun and Lockwood, 2003; Okabayashi et al., 2004). Individuals who are married or live in comparable relationships have been found to be happier and have better mental and physical health than singles (Gove et al., 1983; Holt-Lunstad et al., 2008; Kolves et al., 2012). This association may even become stronger with increasing age (Kansky, 2018). Several studies have also shown that suicidal ideation, depression, suicidal behavior, alcohol abuse, and other mental health issues are more frequent in divorced than married individuals (Stack, 1990, 1992, 2000; Wyder et al., 2009; Batterham et al., 2014). Whereas mental health problems related to a lack or loss of marriage may manifest themselves differently in men and women (i.e., women report more depression and men report more substance abuse), it has been demonstrated that the benefits of being in a marriage-like relationship are similar for men and women (Simon, 2002; Segrin et al., 2003; Simon and Barrett, 2010; Kansky, 2018). Furthermore, married individuals have been found to have lower suicide rates than divorced and widowed individuals as well as individuals who never got married (Corcoran and Nagar, 2010). It has been speculated in scientific literature that relationships have a protective effect, because they provide increased personal satisfaction by satisfying individuals’ needs for social integration and support (Gove et al., 1983; Coyne and DeLongis, 1986; Stack, 2000), and reduce harmful stress (Coyne and DeLongis, 1986; Markey et al., 2007), and unhealthy behaviors (Whitton et al., 2013).

More recent research, however, has highlighted that the association between being in a romantic relationship and mental health is more complex. Whereas romantic relationships can be a protective factor with regards to mental health, they can also be the source of immense personal distress (Coyne and DeLongis, 1986; Kiecolt-Glaser and Newton, 2001; Kazan et al., 2016). In several studies, relationship conflicts or unhappiness in a romantic relationship were associated with psychological distress and low life satisfaction (Glenn and Weaver, 1981; Whisman and Uebelacker, 2006; Carr et al., 2014), higher levels of anxiety and depression (Antonucci et al., 2001; Gallo et al., 2003; Leach et al., 2013; Santini et al., 2015), a higher incidence of psychiatric disorders, in particular major depression (Lapate et al., 2014), and higher risk of non-suicidal self-injury (Levesque et al., 2021), and suicide (Whisman and Uebelacker, 2006; Logan et al., 2011; Bagge et al., 2013; Santini et al., 2015). Till et al. (2017) compared singles to individuals in happy and unhappy relationships in a cross-sectional study and found that singles had significantly lower scores on suicidal ideation, hopelessness, and depression as compared to individuals in unhappy relationships, but significantly higher scores as compared to individuals in happy relationships. Similarly, differences in depression, anxiety, insomnia, psychological quality of life, well-being, and perceived distress have also been found between singles and individuals in good and bad quality relationships in a recent study in Austria, with individuals in good quality relationships having the best and individuals in poor quality relationships having the worst mental health outcomes (Pieh et al., 2020b). One reason for this finding may be that unsatisfying relationships are often characterized by interpersonal conflict, arguments, and domestic violence (Kazan et al., 2016). In several studies, domestic violence has been identified as an essential predictor for suicide risk and self-harming behavior, particularly in females (Levesque et al., 2010; Kavak et al., 2018; Rahmani et al., 2019). These types of relationships can quickly create an environment of violence and abuse, which can render an individual helpless and hopeless, exacerbating vulnerability and increasing suicide risk (Kazan et al., 2016).

Another relationship-related factor that may be important for mental health is the level of commitment in a relationship, which is commonly defined as the intention to maintain a relationship over time (Stanley et al., 2010). In several studies, individuals in relationships with high commitment reported greater well-being than those in relationships with low commitment (Kansky, 2018). Furthermore, several studies found that married men and women are happier and have lower levels of distress than cohabiting, but unmarried, individuals, which scholars have attributed to increased financial satisfaction and overall health in married individuals; in contrast, there were no differences reported in terms of suicidal ideation (Stack and Eshleman, 1998; Whisman and Uebelacker, 2006). It remains unclear, whether the same benefits to mental health found in married individuals extend to other, less committed forms of romantic relationships such as dating casually, dating exclusively, or being engaged (Whitton et al., 2013). This may also depend on whether a couple is also caring for a child. Evidence on the impact of parenthood on mental health is inconclusive, with diverging reports of whether parents have better or worse mental health outcomes (Umberson et al., 2010; Simon and Caputo, 2019). Many authors have pointed out that mental health in parents may be determined by several additional factors (Umberson et al., 2010; Simon and Caputo, 2019), including relationship status and family structure (Hannighofer et al., 2017), but research on this topic is relatively scarce.

Another factor that has emerged recently and may affect both, individuals’ romantic relationship and mental health, is the COVID-19 pandemic, including hardship created by pandemic-related governmental restrictions, physical distancing, and stay-at-home orders. A worsening of mental health during the pandemic has been found in many countries, particularly among women, young people, and individuals in health care professions (Biddle et al., 2020; Czeisler et al., 2020; O’Connor et al., 2021; Ueda et al., 2021; Niederkrotenthaler et al., 2022). Also caregivers of children have been found to be particularly affected by hardship faced during the COVID-19 pandemic, resulting in greater mental distress in individuals living with children in one household (Okabayashi et al., 2004; Pierce et al., 2020; Niederkrotenthaler et al., 2022). Takaku and Yokoyama (2021a,b) found an increase in anxiety and physical domestic violence in wives affected by pandemic-related school closure, but no impact with regards to marital relationship and marital quality. In another survey, however, 17.5% of respondents reported that their relationships has worsened during the COVID-19 pandemic (Biddle et al., 2020). Studies investigating whether individuals’ mental health has been affected by COVID-19 differently depending on relationship status, satisfaction, commitment, or family structure are currently lacking.

Overall, studies that investigated the role of relationship-related factors in mental health so far are relatively diverse and heterogeneous, but the majority of them used convenience samples and/or relatively broad mental health indicators such as “distress” or “happiness.” Some studies also focused only on specific age groups such as older adults or college students (Antonucci et al., 2001; Gallo et al., 2003; Whitton et al., 2013; Carr et al., 2014), which may yield different results. Thus, the current study had two aims: First, we aimed to replicate the study by Till et al. (2017) on relationship satisfaction and suicide risk factors and extend it by using a large population-based sample and assessing a broader range of relevant mental health outcomes. In order to account for the current situation involving the COVID-19 pandemic, we also explored differences between singles and individuals in happy and unhappy relationships with regard to COVID-19-related changes in metal health. Second, we aimed to explore differences in terms of mental health in individuals currently in a relationship with regards to relationship commitment and family structure. We hypothesized that scores for mental health outcomes will be more favorable in individuals in happy romantic relationships, less favorable in singles, and the least favorable in individuals in unhappy relationships. Furthermore, we hypothesized that mental health outcome scores will be highest (i.e., least favorable) in individuals who are currently in relationships with low commitment and living with a child in the same household.

## Materials and Methods

The data analyzed in the current work were collected as part of a larger project aiming to explore and monitor mental health in Austria during the first 9 months of the COVID-19 pandemic and identify risk groups for mental-ill health. For this project, a repeated cross-sectional online quota survey was conducted every 3 weeks from April to December 2020 (Niederkrotenthaler et al., 2022). Three of the twelve waves (dates: September 18–29, October 9–21, October 30–November 11) focused particularly on the role of romantic relationships and relationship satisfaction in mental health. The data collected in these particular waves were used in the current study.

### Participants

Data were collected in three waves, and each wave consisted of approximately 1,000 participants who were representative of the population in Austria for individuals of 16 years and older based on quotas in terms of gender (i.e., male, female, diverse), age (age groups were: 16–29, 30–39, 40–49, 50–59, 60–69, 70+ years), region (i.e., all nine federal states of Austria), and education (i.e., below high school, high school, and college). Representative data of younger individuals were unavailable. Furthermore, in order to increase the sample’s representativeness of the Austrian population, the dataset was weighted in terms of combinations across all four variables by using RIM weighting (i.e., random iterative method).

All participants were recruited by a professional marketing company, *Ipsos*, from an online panel consisting of 30,000 registered members via emails sent out based on remaining quotas and computerized individual response probabilities. Of the 20,221 panel members who were invited to participate in the study, 4,370 individuals accepted the invitation and started the survey. Whereas 1,010 respondents were screened out because of completed quotas and 348 respondents ended their participation before finishing the survey, 3,012 participants completed the entire survey and were included in the statistical analysis.

### Measures

#### Suicidal Ideation

We used the German short form (Kliem and Brähler, 2015) of the Beck Scale for Suicidal Ideation (BSS; Beck and Steer, 1993) to assess suicidal ideation in the past week. This self-report measure consists of five items rating the intensity of suicidality on a 3-point scale from 0 to 2 (e.g., “*I have no/a weak/a moderate to strong wish to die*”). A mean score was calculated across all items, with high scores indicating high suicidal ideation (score range: 0–2; Cronbach’s α = 0.86).

#### Depression

The Patient Health Questionnaire (PHQ-9) by Kroenke et al. (2001) consisting of nine items that rate depressive symptoms in the last 2 weeks (e.g., “*Feeling down, depressed, or hopeless*”) on a scale from 0 (*not at all*) to 3 (*nearly every day*) was used to assess depression. This scale or a short version of this scale was used in multiple other studies that assessed depression during the COVID-19 pandemic (e.g., Czeisler et al., 2020; Olagoke et al., 2020; Pieh et al., 2020a,b; Ueda et al., 2020; O’Connor et al., 2021). A mean score was calculated across all items, with high scores indicating a high level of depression (score range: 0–3; Cronbach’s α = 0.91).

#### Anxiety

In order to assess respondents’ anxiety, we used the anxiety subscale of the Hospital Anxiety and Depression Scale (HADS) by Zigmond and Snaith (1983), which consists of seven items that rate the severity of anxiety in the past week from 0 to 3 (e.g., “*Worrying thoughts go through my mind a great deal of the time/a lot of the time/from time to time but not too often/only occasionally*”). A mean score was calculated across all items, with high scores indicating greater anxiety (score range: 0–3; Cronbach’s α = 0.83).

#### Psychological and Physical Violence

After a brief definition of psychological and physical violence, respondents rated their experience of psychological and physical violence in the last 2 weeks with one single item, respectively (“Did you experience any (A) psychological/(B) physical violence by your partner or any other family member in the last 2 weeks?”), on a scale from 1 (*not at all*) to 5 (*very much*).

#### Changes in Mental Health During the COVID-19 Pandemic

In order to assess changes in suicidal ideation compared to before COVID-19, we asked respondents rate the frequency and amount of their current suicidal ideation as compared to before the pandemic on a scale from 1 (*much smaller or much less frequent*) to 5 (*much greater or much more frequent*), including one category stating “*I have no suicidal ideation*”. Furthermore, changes in anxiety compared to before COVID-19 were assessed with one item rating the frequency/amount of their current anxiety as compared to before the pandemic on a scale from 1 (*much smaller or much less frequent*) to 5 (*much greater or much more frequent*).

#### Relationship Status

Consistent with a previous research, we asked respondents to indicate whether they are currently in a romantic relationship (*yes* = 1, *no* = 0).

#### Relationship Satisfaction

The Relationship Assessment Scale by Hendrick (1988) was used to assess relationship satisfaction. This self-report measure consists of seven items (e.g., “In general, how satisfied are you with your relationship?”) that are rated on a 5-point scale ranging from 1 (*low satisfaction*) to 5 (*high satisfaction*). This scale was only completed by participants in romantic relationships. A mean score was calculated across all items, with high scores indicating high relationship satisfaction (score range: 1-5; Cronbach’s α = 0.92).

#### Civil Status

We asked respondents to indicate their civil status by selecting one of the following five categories: Single, partnered, married, widowed, or other. If participants selected “other,” then they were asked to elaborate on their civil status in an open-ended response. Since the vast majority of respondents in the “other” category indicated that they were either divorced or lived separately from their partner (without being officially divorced), we added an additional category for “divorced” to civil status. The remaining respondents in the “other” category were categorized as *single*, *partnered*, *married*, *widowed*, or *divorced* based on their respective responses (e.g., erroneously selecting “other”, but writing “single” in the open-ended response).

#### Child in the Same Household

We asked respondents to indicate whether there is currently a child of school age or younger living in their household (*yes* = 1, *no* = 0). According to Austrian law, school attendance is compulsory for 9 years and starts on September 1 following the child’s sixth birthday (Austrian Federal Ministry of Education, Science and Research, 2021).

### Data Analysis

In order to test whether there are differences in mental health between singles, individuals in happy romantic relationships, and individuals in unhappy romantic relationships, we split our sample into one group with high relationship satisfaction (*n* = 1,018, *M* = 4.81, *Md* = 4.86, *IQR* = 0.43, *Min* = 4.43, *Max* = 5.00) and one group with low relationship satisfaction (*n* = 998, *M* = 3.50, *Md* = 3.71, *IQR* = 1.14, *Min* = 1.00, *Max* = 4.29) by the median (*Md* = 4.43) of the Relationship Assessment Scale scores. Relationship satisfaction in the group labeled as “low relationship satisfaction” was consistent with scores found in individuals seeking marital and family therapy at a marriage and family clinic, and relationship satisfaction in the group labeled as “high relationship satisfaction” was consistent with scores in happily married or recently dating couples (Inman-Amos et al., 1994; Hendrick et al., 1998).

These two groups (high relationship satisfaction: 33.8%; low relationship satisfaction: 33.1%) were compared to those respondents who were currently not in a romantic relationship (*n* = 996, 33.1%). This approach is consistent with previous research (Till et al., 2017). Mean differences between the three groups were examined for all mental health outcomes with *F*-tests from analyses of variance (ANOVAs), and individual group differences were tested for significance with Bonferroni-corrected contrast tests. Table 1 provides an overview of socio-demographic characteristics of participants, and see Table 2 for an overview of the groups’ mental health outcomes along with results from ANOVAs. Of note, males and younger individuals had a higher probability of not currently being in a romantic relationship.

**TABLE 1:** Overview of socio-demographic characteristics of all participants (*n* = 3,012).

Characteristic	Total	No relationship (*n* = 996)	High relationship satisfaction (*n* = 1,018)	Low relationship satisfaction (*n* = 998)	χ^2^
**Gender**					
Male	1,456 (48.4)	432 (43.3)	530 (52.1)	495 (49.6)	16.42**^a^
Female	1,551 (51.5)	562 (56.5)	487 (47.8)	501 (50.2)	
Other genders	5 (0.2)	2 (0.2)	1 (0.1)	2 (0.2)	
**Age group**					
16–29	624 (20.7)	330 (33.2)	146 (14.3)	149 (14.9)	196.23***^b^
30–39	470 (15.6)	115 (11.5)	156 (15.3)	200 (20.0)	
40–49	590 (19.6)	184 (18.4)	172 (16.9)	235 (23.5)	
50–59	487 (16.2)	135 (13.5)	194 (19.0)	159 (15.9)	
60–69	376 (12.5)	121 (12.2)	130 (12.8)	124 (12.4)	
70+	463 (15.4)	111 (11.1)	221 (21.7)	132 (13.2)	
**Education**					
Below high school	2,243 (74.5)	756 (75.9)	756 (75.2)	722 (72.3)	9.21^a^
High school	414 (13.8%)	144 (14.4)	125 (12.3)	146 (14.6)	
College/university	355 (11.8%)	96 (9.7)	128 (12.6)	130 (13.1)	
**Relationship status**					
Relationship	2,016 (66.9)	0 (0.0)	1018 (100)	998 (100)	3012.00***^c^
No relationship	996 (33.1)	996 (100)	0 (0.0)	0 (0.0)	

Values are weighted absolute (n) and relative (%) frequencies of participants’ socio-demographic characteristics as well as χ^2^ values from χ^2^ tests testing group differences, **p < 0.01; ***p < 0.001 (two-tailed). ^a^df = 4. ^b^df = 10. ^c^df = 2.

**TABLE 2:** Mental health among participants with high and low relationship satisfaction and participants currently not in a relationship (*n* = 3,012).

	No relationship (*n* = 996)	High relationship satisfaction (*n* = 1,018)	Low relationship satisfaction (*n* = 998)		
Mental Health Outcomes	*M* (95% CI)	*M* (95% CI)	*M* (95% CI)	*F*	η_*p*_^2^
Suicidal ideation (α = 0.86)	0.16 (0.14–0.18)	**0.04 (0.03–0.05)**	0.14 (0.12–0.16)	36.53***^a^	0.024
Suicidal ideation compared to before pandemic	2.62 (2.46–2.77)	2.32 (2.08–2.56)	2.71 (2.59–2.84)	3.99*^b^	0.014
Depression (α = 0.91)	0.74 (0.70–0.79)	**0.41 (0.38–0.44)**	0.73 (0.69–0.77)	72.28***^a^	0.046
Anxiety (α = 0.83)	0.76 (0.72–0.79)	**0.49 (0.46–0.52)**	**0.84 (0.81–0.88)**	89.58***^a^	0.056
Anxiety compared to before pandemic	2.81 (2.75–2.87)	**2.66 (2.60–2.72)**	**2.91 (2.86–2.97)**	16.63***^a^	0.011
Psychological violence	1.37 (1.32–1.43)	**1.09 (1.06–1.12)**	**1.58 (1.51–1.64)**	78.71***^a^	0.050
Physical violence	1.20 (1.16–1.24)	**1.05 (1.03–1.07)**	**1.33 (1.28–1.37)**	48.74***^a^	0.031

Values are means (M) with 95% confidence intervals (95% CI) given in parentheses and Cronbach’s alphas (α) of the variables as well as F values and effect sizes (η_p_^2^) of analyzes of variance estimated with weighted data, *p < 0.05; ***p < 0.001 (two-tailed). Significant differences compared to the group consisting of participants not currently in a relationship as indicated by significant contrast tests (p < 0.05) are bold. ^a^df_1_ = 2, df_2_ = 3,004. ^b^df_1_ = 2, df_2_ = 544.

In order to test whether there are differences in mental health with regards to relationship commitment and family structure, we categorized respondents who identified as “*partnered*” or “*married*” as individuals in relationships with high commitment (*n* = 1,773; 87.9%) and respondents who indicated that they are currently in a romantic relationship, but identified as “*single*”, as individuals in relationships with low commitment (*n* = 220; 10.9%). All remaining participants (*n* = 24; 1.2%) were excluded from this part of the analysis. Furthermore, of the included 1,993 respondents, *n* = 593 (29.8%) indicated that they were currently living in a household with a child of school age or younger, whereas *n* = 1,400 respondents (70.2%) indicated that this was currently not the case. See Table 3 for an overview of mental health outcomes stratified by relationship commitment and family structure. Differences in mental health with regards to relationship commitment and family structure were examined with ANOVAs. We used mental health outcomes as dependent variables and relationship commitment and family structure as fixed factors, and individual group differences were tested for significance with Bonferroni-corrected contrast tests. See Table 4 for findings from ANOVAs for mental health with regard to relationship commitment and family structure. All analyzes were controlled for wave number (i.e., time) by including a dummy variable for each wave, age group, and gender (with one dummy variable for females and one for other gender). Previous studies have found age and gender to be associated with relationship satisfaction (Jose and Alfons, 2007; Jackson et al., 2014; Kansky, 2018), and COVID-19-related measures (Ueda et al., 2020; O’Connor et al., 2021; Niederkrotenthaler et al., 2022). Furthermore, gender and age were not equally distributed across the study groups (see Table 1).

**TABLE 3:** Mental health among participants with relationships stratified by relationship commitment and family structure (*n* = 1,993).

	High commitment (*n* = 1,773)	Low commitment (*n* = 220)	Child in household (*n* = 593)	No child in household (*n* = 1,400)
	
Mental Health Outcomes	*M* (95% CI)	*M* (95% CI)	*M* (95% CI)	*M* (95% CI)
Suicidal ideation	0.08 (0.07–0.10)	0.14 (0.09–0.18)	0.10 (0.07–0.12)	0.09 (0.07–0.10)
Suicidal ideation compared to before pandemic	2.57 (2.45–2.70)	2.68 (2.39–2.97)	2.61 (2.42–2.81)	2.58 (2.44–2.72)
Depression	0.54 (0.51–0.56)	0.80 (0.71–0.89)	0.69 (0.64–0.74)	0.51 (0.48–0.54)
Anxiety	0.65 (0.62–0.67)	0.87 (0.79–0.95)	0.81 (0.76–0.86)	0.61 (0.58–0.64)
Anxiety compared to before pandemic	2.80 (2.75–2.84)	2.72 (2.59–2.85)	2.87 (2.79–2.95)	2.75 (2.70–2.80)
Psychological violence	1.30 (1.27–1.34)	1.57 (1.43–1.71)	1.52 (1.44–1.60)	1.25 (1.22–1.29)
Physical violence	1.16 (1.14–1.19)	1.38 (1.27–1.49)	1.30 (1.24–1.36)	1.14 (1.11–1.16)

*Table entries are means (M) with 95% confidence intervals (95% CI) given in parentheses estimated with weighted data.*

**TABLE 4:** Findings from analyzes of variance for mental health with regard to relationship commitment and family structure.

Mental health outcomes	Relationship commitment		Family structure		Relationship commitment × family structure	
	*F*	η_*p*_^2^	*F*	η_*p*_^2^	*F*	η_*p*_^2^
Suicidal ideation^a^	17.28***	0.009	17.04***	0.008	31.25***	0.015
Suicidal ideation compared to before pandemic^b^	0.07	0.000	0.23	0.001	0.01	0.000
Depression^a^	9.49**	0.005	3.46	0.002	2.18	0.001
Anxiety^a^	6.84*	0.003	4.74*	0.002	0.23	0.000
Anxiety compared to before pandemic^a^	4.14*	0.002	0.02	0.000	0.70	0.000
Psychological violence^a^	17.10***	0.009	23.09***	0.011	7.50**	0.004
Physical violence^a^	19.83***	0.010	18.88***	0.009	8.42**	0.004

Table entries are F values and effect sizes (η_p_^2^) of analyzes of variance of mental health outcomes with regard to relationship commitment (high vs. low commitment), family structure (child vs. no child in household), and interactions between these factors estimated with weighted data, *p < 0.05; **p < 0.01; ***p < 0.001 (two-tailed). ^a^df_1_ = 1, df_2_ = 1,991. ^b^df_1_ = 1, df_2_ = 324.

IBM SPSS version 26 was used for all statistical analyzes. Visualizations were done with IBM SPSS version 26 and GIMP version 2.10.28.

## Results

### Mental Health With Regards to Relationship Status and Relationship Satisfaction

The analyzes of variance revealed that there were significant differences between singles, individuals in happy romantic relationships, and individuals in unhappy romantic relationships with regards to all mental health outcomes (see Table 2). Results from contrast tests indicated that mental health outcomes were significantly more favorable across all scores among individuals with high relationship satisfaction than among those with low relationship satisfaction and among individuals who were currently not in a relationship (except for the scores of suicidal ideation compared to before the pandemic between individuals with high relationship satisfaction and those not in a relationship). Furthermore, anxiety, anxiety compared to before the pandemic, psychological violence, and physical violence were higher among individuals with low relationship satisfaction as compared to those who were not in a relationship (see Table 2). The sizes of the found group differences were either small or in the small to medium range, with η_*p*_^2^ ranging from 0.011 to 0.056.

### Mental Health With Regards to Relationship Commitment and Family Structure

**FIGURE 1:**
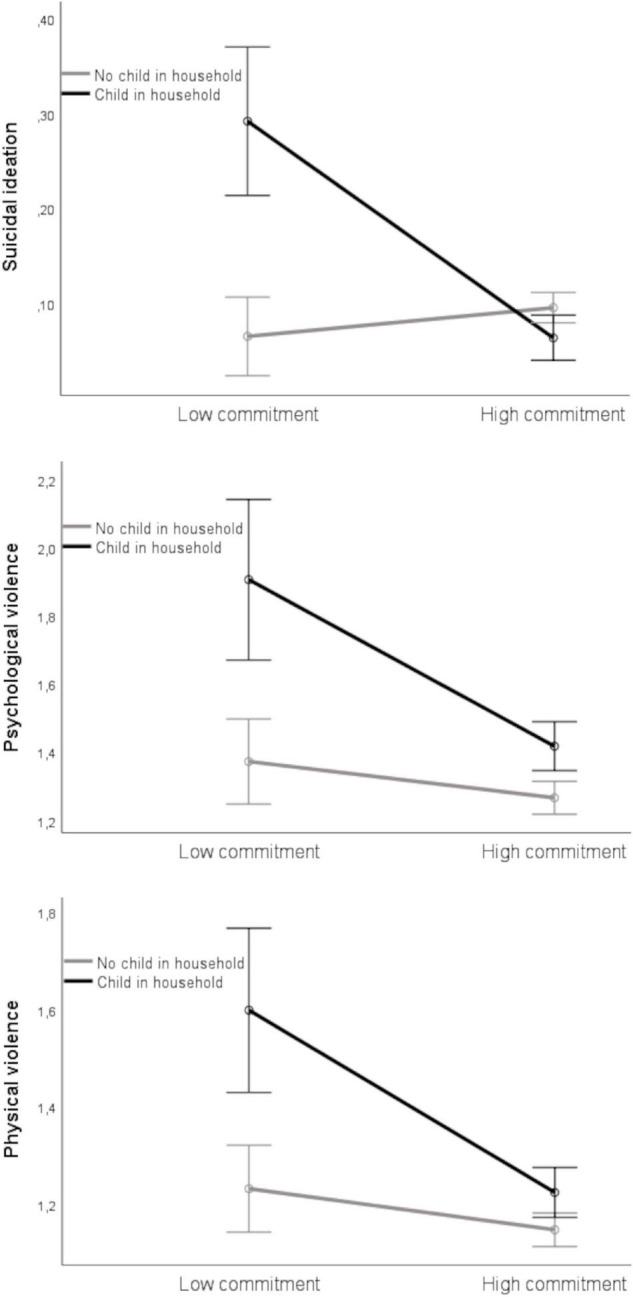
Suicidal ideation, psychological violence, and physical violence by relationship commitment and family structure.

The analyzes of variance (see Table 4) revealed a significant interaction effect of relationship commitment and family structure for suicidal ideation, psychological violence, and physical violence. Results of contrast tests indicated that suicidal ideation was higher and the experience of psychological and physical violence was more frequent in individuals in relationships with low commitment and with a child in the household as compared to all other groups (all *p* < 0.001). An illustration and overview of these group differences can be found in Figure 1 and Table 3. Furthermore, depression was higher among individuals in relationships with low commitment than those with high commitment. In terms of anxiety, scores were significantly higher for individuals in relationships with low than those with high commitment, and scores were higher for individuals with a child in the household as compared to no child (but there was no significant interaction effect of relationship commitment and family structure). In contrast, scores for anxiety compared to before the pandemic were significantly higher for individuals in high commitment relationships than for those in relationships with low commitment. The sizes of all group differences were small, with η_*p*_^2^ ranging from 0.002 to 0.015 (see Table 4).

### Sensitivity Analysis

We performed a sensitivity analysis to assess whether patterns differed if unweighted instead of weighted data were used. Tables providing results of descriptive statistics and analyzes of variance with unweighted data can be found in Supplementary Tables 1–3. Results of analyzes with unweighted data were similar to those with weighted data. The significant main effect of commitment originally found for anxiety compared to before the pandemic was not significant in the sensitivity analysis, but was close to the boundaries of statistical significance. Furthermore, we performed a sensitivity analysis to test whether patterns differed if respondents who indicated that they are currently in a romantic relationship, but identified their civil status as “*divorced*” or “*widowed*” (*n* = 24) were included in the analysis and coded as individuals in relationships with low commitment. Results of analyzes with and without these 24 respondents were almost identical (data available upon request).

## Discussion

### Relationship Status and Relationship Satisfaction

The current study aimed to replicate and expand on findings from a previous study (Till et al., 2017) with a large sample representative of the Austrian population in terms of gender, age, education, and region of residence and provide additional empirical evidence on differences in mental health with regards to relationship status and relationship satisfaction. The results showed that mental health outcomes were less favorable among singles than among individuals in happy romantic relationships, but more favorable than among those in unhappy relationships, confirming the findings of Till et al. (2017) in a nationally representative sample. These findings are also consistent with many other studies that found that mental health is better in individuals who are currently in a romantic relationship as compared to singles (Gove et al., 1983; Stack, 1990, 1992, 2000; Holt-Lunstad et al., 2008; Wyder et al., 2009; Kolves et al., 2012; Batterham et al., 2014; Carr et al., 2014; Kansky, 2018) and in individuals in happy or high quality relationships as compared to those in unhappy or low quality relationships (Gallo et al., 2003; Logan et al., 2011; Bagge et al., 2013; Leach et al., 2013). The current findings highlight that individuals in happy romantic relationships report beneficial mental health, including comparatively low scores for suicidal ideation, depression, anxiety, and psychological and physical violence; however, being dissatisfied with one’s relationship seems to be a major indicator for poor mental health. In fact, scores for anxiety as well as psychological and physical violence were significantly higher among individuals in relationships with comparatively lower satisfaction than in singles. This is consistent with findings from previous studies highlighting similar differences in mental health between singles and individuals in good and bad quality relationships (Pieh et al., 2020b) and showing that relationship problems were associated with increased suicide risk (Stack, 2000). Interpersonal problems were also the most frequently mentioned reason for a suicidal crisis in suicide online message boards (Niederkrotenthaler and Till, 2019).

As discussed in previous studies (Till et al., 2017), individuals living in unhappy and maybe even hostile or violent relationships may tend to experience a feeling of social alienation and perceived burdensomeness (Kazan et al., 2016), which has been found to be associated with increased suicide risk (Joiner, 2005; Van Orden et al., 2010). It may also be the case that, once an individual develops a mental illness, their chances of experiencing a deterioration of relationship quality or satisfaction increase. In fact, individuals with common mental illnesses have been found to have a greater risk of marital dissolution and they are less likely to get married or enter new marriages (Teitler and Reichman, 2008; Mojtabai et al., 2017). Thus, relationship satisfaction may be a marker of comparatively good mental health and low suicide risk. This also highlights that it may be useful to collect data on relationship satisfaction in addition to relationship status when suicide risk is assessed (Till et al., 2017). In order to assess any causal link between relationship status and mental health, it is necessary to apply longitudinal research designs in the future.

### Relationship Commitment and Family Structure

The current study also aimed to explore whether there are differences in mental health of individuals who are currently in a relationship with regards to relationship commitment and family structure. The results showed that depression was higher in individuals in relationships with low commitment as compared to those in high commitment relationships. Furthermore, suicidal ideation as well as psychological and physical violence in the relationship were particularly high among individuals who were both, in a relationship with low commitment and with a child in the same household. It may be the case that having parental responsibilities, which can be very stressful (Jennings and Dietz, 2007), but not a partner or spouse who is committed to the relationship, may be reflected in frequent conflicts in the relationship and a deterioration of emotional well-being. Consistent with this notion, low relationship commitment was associated with higher levels of relationship conflict and distress and lower levels of emotional well-being and happiness in several previous studies (Stack and Eshleman, 1998; Knee et al., 2004; Whisman and Uebelacker, 2006; Kansky, 2018). Furthermore, anxiety was higher in the current study when individuals were in a relationship with low commitment (as compared to a high commitment relationship) or currently living with a child in the same household (as compared to no child in the household). Both factors may contribute to stress in the relationship (Stack and Eshleman, 1998; Whisman and Uebelacker, 2006; Simon and Caputo, 2019), which has been found to be associated with higher levels of anxiety (Naerde and Sommer Hukkelberg, 2020). It could also be the case that individuals with higher levels of anxiety are also more hesitant to commit to a relationship (Etcheverry et al., 2012).

### Romantic Relationships and Mental Health With Regards to COVID-19

Individuals in happy relationships had a lower probability of experiencing an increase in suicidal ideation or anxiety during the COVID-19 pandemic than individuals in unhappy relationships. Furthermore, the probability of experiencing an increase in anxiety during the COVID-19 pandemic was higher in singles when compared to individuals in happy relationships and lower when compared to those in unhappy relationships. This is consistent with findings by Pieh et al. (2020b) highlighting that being in a good quality romantic relationship predicted good mental health during the COVID-19 pandemic. It seems that having satisfactory support by a partner or spouse during stressful times in one’s life, particularly a time in which people spend a lot of time at home due to lockdowns, stay-at-home orders, and working from home policies, may help to prevent an increase in suicidal ideation or anxiety.

Regarding relationship commitment and family structure, scores for anxiety compared to before the COVID-19 pandemic were higher in individuals in relationships with high commitment than those with low commitment. A possible explanation for this counterintuitive finding may be that there was some kind of floor effect. Anxiety levels in individuals in relationships with high commitment may have been already very low before the pandemic, which may have prevented any further reduction in anxiety. Another possible explanation may be related to (expected) conflicts during the pandemic. Several studies found an increase in conflicts and domestic violence within a household or family during the COVID-19 pandemic (e.g., Feder et al., 2021; Takaku and Yokoyama, 2021a; Niederkrotenthaler et al., 2022). However, many couples in relationships with low commitment may not live in the same household and were therefore maybe less affected by lockdowns and stay-at-home orders in terms of increases in interpersonal conflicts, or worries about upcoming interpersonal conflicts, which may have positively impacted their anxiety as compared to individuals in relationships with high commitment.

### Relevance and Practical Significance

The sizes of most identified differences in mental health with regards to relationship status, relationship satisfaction, relationship commitment and family structure were all relatively small (see Tables 2, 4). Furthermore, for all explanatory variables, the identified group differences may have been statistically significant, but they were all numerically small (see Tables 2, 3). As discussed in the literature, small effect sizes and small numerical differences are sometimes considered an indicator for a lack of clinical or practical significance (LeFort, 1993; Ferguson, 2009; Ranganathan et al., 2015). However, several scholars have highlighted that clinical or practical significance cannot be evaluated just based on effect sizes and numerical differences alone (LeFort, 1993; Ferguson, 2009; Ranganathan et al., 2015; Anvari et al., 2021). In fact, in the context of the high number of individuals living in romantic relationships (with various degrees of satisfaction with their relationship), the significant mean differences found in the current study may have important implications for mental health in the population. Considering that romantic relationships are an integral part of our lives and affect us on a daily basis (Whisman and Baucom, 2012; Kazan et al., 2016), even small group differences in mental health may be of practical relevance (Anvari et al., 2021). Furthermore, relationship satisfaction in the group labeled as “low relationship satisfaction” was consistent with scores found in individuals seeking marital and family therapy at a marriage and family clinic (Hendrick et al., 1998). Differences in mental health may be even greater if compared between happily married couples and individuals living in disrupted or violent romantic relationships.

### Strengths and Limitations

The use of a large quota-based sample that was representative of the Austrian population in terms of gender, age, education, and region of residence was a strength of the current study. There are also some limitations. First, we cannot infer causality regarding the identified differences in mental health due to the cross-sectional design of this study. Furthermore, whereas anxiety was measured in the current study, we did not assesses what respondents were specifically anxious about or whether their anxiety was somehow related to their relationship at all. Moreover, both COVID-19-related measures were single-item assessments and particularly prone to recall bias. Another limitation was that we did not explicitly explain the term “romantic relationship” in the survey, and respondents did not have the opportunity to indicate if they were currently living in multiple relationships, or if they defined themselves as aromantic. We also did not assess the sexual identity of the participants and their partners, which may be important for mental health differences with regard to romantic relationships. There are currently no clear patterns about these associations among gender and sexual minority groups. Finally, gender and age were not equally distributed across study groups, which may have biased the results, but we have controlled for both variables in our analyzes.

## Conclusion

The findings of the current study confirm evidence of previous studies (Kiecolt-Glaser and Newton, 2001; Carr et al., 2014; Till et al., 2017) that several clinically relevant mental health outcomes are reflected in individual demographics related to romantic relationships such as relationship status, relationship satisfaction, relationship commitment, and family structure. Our results indicate that singles may have less favorable scores of mental health than individuals in happy relationships, but better mental health than individuals in unhappy relationships. This may indicate that being single may be more beneficial for one’s mental health than staying in an unhappy relationship (Till et al., 2017), but a definite statement cannot be made based on the cross-sectional data of the current study. Furthermore, mental health tended to be worse in individuals who were in a relationship with low commitment and lived with a child in the household as compared to individuals in relationships with high commitment or without a child in the household. Relationship distress may be high when individuals are confronted with parental responsibilities (Jennings and Dietz, 2007) without having the support of a highly committed partner or spouse (Stack and Eshleman, 1998; Knee et al., 2004; Whisman and Uebelacker, 2006; Kansky, 2018). Romantic relationships need to be more emphasized in psychiatric research, and clinicians may want to take information on the quality of a patient’s romantic relationships into account when assessing their suicide risk or mental health, which is a core component in many psychological techniques, including crisis intervention. Furthermore, partner counseling should be made more available and accessible to couples who want to work on their relationship but need help or assistance in this process, particularly in the current aftermath of the COVID-19 pandemic, which further increased distress in families (Pierce et al., 2020). More research, however, on the role of romantic relationships in individuals’ mental health, is necessary to gain further insights into potential risk and protective factors. In particular, longitudinal studies are needed to identify causal effects.

## Data Availability Statement

The raw data supporting the conclusions of this article will be made available by the authors, without undue reservation.

## Ethics Statement

Ethical review and approval was not required for the study on human participants in accordance with the local legislation and institutional requirements. Written informed consent from the participants’ legal guardian/next of kin was not required to participate in this study in accordance with the national legislation and the institutional requirements.

## Author Contributions

BT conceived and designed the study, performed data collection, analyzed the data, and wrote the first draft of the manuscript. TN supervised and provided important intellectual content in revising the manuscript. Both authors contributed to the article and approved the submitted version.

## Conflict of Interest

The authors declare that the research was conducted in the absence of any commercial or financial relationships that could be construed as a potential conflict of interest.

## Publisher’s Note

All claims expressed in this article are solely those of the authors and do not necessarily represent those of their affiliated organizations, or those of the publisher, the editors and the reviewers. Any product that may be evaluated in this article, or claim that may be made by its manufacturer, is not guaranteed or endorsed by the publisher.
