# Supplementary material for: Romantic Relationships and Mental Health During the COVID-19 Pandemic in Austria: A Population-Based Cross-Sectional Survey
Source: Front Psychol. 2022 Apr 27;13:857329. doi: 10.3389/fpsyg.2022.857329 (PMC9093599; doi:10.3389/fpsyg.2022.857329)
Supplement: Supplementary file 1 [file Table_2.PDF]

Supplementary Table 1

Descriptive Statistics for Mental Health among Participants with High and Low Relationship Satisfaction and Participants Currently Not in a Relationship (n = 3,012)

	No Relationship (n = 988)	High Relationship Satisfaction (n = 1,034)	Low Relationship Satisfaction (n = 990)	
Mental Health Outcomes	M (95% CI)	M (95% CI)	M (95% CI)	F (df_1, df_2)
Suicidal ideation (α = .85)	0.16 (0.14 – 0.18)	**0.04 (0.03 – 0.05)**	0.14 (0.11 – 0.16)	38.26^{***a}
Suicidal ideation compared to before pandemic	2.63 (2.48 -2.79)	2.30 (2.07 – 2.54)	2.70 (2.58 – 2.83)	4.11^{*b}
Depression (α = .91)	0.74 (0.70 – 0.78)	**0.40 (0.37 – 0.43)**	0.72 (0.68 – 0.76)	76.01^{***a}
Anxiety (α = .83)	0.75 (0.71 – 0.79)	**0.49 (0.46 – 0.51)**	**0.84 (0.81 – 0.88)**	93.84^{***a}
Anxiety compared to before pandemic	2.80 (2.74 – 2.86)	**2.65 (2.59 – 2.71)**	**2.91 (2.86 – 2.97)**	17.51^{***a}
Psychological violence	1.37 (1.31 – 1.42)	**1.09 (1.06 – 1.11)**	**1.57 (1.50 – 1.63)**	77.88^{***a}
Physical violence	1.19 (1.15 -1.23)	**1.04 (1.02 – 1.06)**	**1.32 (1.28 – 1.37)**	49.98^{***a}

Values are means (M) with 95% confidence intervals (95% CI) given in parentheses and Cronbach's alphas (α) of the variables as well as F values with degrees of freedom (df_1, df_2) given in parentheses from analyses of variance estimated with unweighted data, * $p < .05$; ** $p < .01$; *** $p < .001$ (two-tailed). Significant differences compared to the group consisting of participants not currently in a relationship as indicated by significant contrast tests ($p < .05$) are bold.

$^{a}df_1 = 2$, $df_2 = 3004$

$^{b}df_1 = 2$, $df_2 = 544$

Supplementary Table 2

Descriptive Statistics for Mental Health among Participants with Relationships Stratified by Relationship Commitment and Family Structure (n = 2,000)

	High Commitment (*n* = 1,783)	Low Commitment (*n* = 217)	Child in Household (*n* = 566)	No Child in Household (*n* = 1,434)
Mental Health Outcomes	*M* (95% CI)	*M* (95% CI)	*M* (95% CI)	*M* (95% CI)
Suicidal ideation	0.08 (0.07 – 0.09)	0.13 (0.09 – 0.17)	0.10 (0.07 – 0.12)	0.08 (0.07 – 0.10)
Suicidal ideation compared to before pandemic	2.56 (2.43 – 2.68)	2.68 (2.39 – 2.98)	2.58 (2.38 – 2.78)	2.58 (2.44 – 2.72)
Depression	0.53 (0.50 – 0.55)	0.80 (0.71 – 0.89)	0.68 (0.64 – 0.73)	0.51 (0.48 – 0.54)
Anxiety	0.64 (0.61 – 0.67)	0.87 (0.78 – 0.95)	0.81 (0.76 – 0.86)	0.61 (0.58 – 0.64)
Anxiety compared to before pandemic	2.79 (2.74 – 2.83)	2.72 (2.59 – 2.86)	2.87 (2.79 – 2.94)	2.75 (2.70 – 2.80)
Psychological violence	1.29 (1.26 – 1.33)	1.58 (1.43 – 1.72)	1.51 (1.43 – 1.59)	1.25 (1.21 – 1.29)
Physical violence	1.16 (1.13 – 1.18)	1.38 (1.27 – 1.49)	1.30 (1.24 – 1.36)	1.13 (1.11 – 1.16)

Table entries are means (*M*) with 95% confidence intervals (95% CI) given in parentheses estimated with unweighted data.

Supplementary Table 3

Findings from Analyses of Variance for Mental Health with regard to Relationship Commitment and Family Structure

**Mental Health Outcomes**	**Relationship Commitment**	**Family Structure**	**Relationship Commitment × Family Structure**
Suicidal ideation[a]	17.13***	15.72***	30.09***
Suicidal ideation compared to before pandemic[b]	0.03	0.44	0.04
Depression[a]	10.83**	3.43	2.62
Anxiety[a]	6.95**	4.86*	0.36
Anxiety compared to before pandemic[a]	3.36	0.00	0.36
Psychological violence[a]	19.10***	22.64***	7.97**
Physical violence[a]	20.28***	18.43***	8.32**

Table entries are F values from analyses of variance of mental health outcomes with regard to relationship commitment (high vs. low commitment), family structure (child vs. no child in household), and interactions between these factors estimated with unweighted data, * $p < .05$; ** $p < .01$; *** $p < .001$ (two-tailed).

[a] $df_1 = 1$, $df_2 = 1{,}991$

[b] $df_1 = 1$, $df_2 = 324$
